# Differences in Maximum Upper and Lower Limb Strength in Older Adults After a 12 Week Intense Resistance Training Program

**DOI:** 10.2478/v10078-011-0086-x

**Published:** 2011-12-25

**Authors:** Nelson Sousa, Romeu Mendes, Catarina Abrantes, Jaime Sampaio

**Affiliations:** 1 Research Center in Sport Health & Human Development, Portugal; 2 University of Trás-os-Montes and Alto Douro, Sport Sciences, Exercise & Health Department, Portugal; 3 University of Beira Interior, Portugal

**Keywords:** aging, upper limbs, lower limbs, muscle atrophy, exercise

## Abstract

The purpose of this study was to identify differences in maximum strength after an intense strength training program, contrasting muscle groups from upper limbs versus lower limbs. The sample consisted of 10 healthy elderly males (age 73±6 years) with independent living. The training program lasted 12 weeks (3 × week, 50 to 80% of 1RM, 2–3 sets, 6 to 12 repetitions). Two muscle groups were analyzed: LOWER (sum of average values of three exercises for the lower limbs) and UPPER (sum of average values of four exercises for the upper limbs). Measurement of 1RM was performed at intervals of 4 weeks by direct methods. Repeated measures ANOVA identified significant differences in muscle groups (F=8.1, p=0.006), time (F=730.0 p=0.000) and also their interaction (F=4.4, p=0.014). The gains in 1RM values were higher for upper limbs. These results may suggest that the muscles of the lower limbs are elicited more frequently and therefore, have a smaller potential to gain strength at older age. The muscles of the upper limbs are in accelerated muscle atrophy and their trainability is probably higher.

## Introduction

The world population is aging rapidly and therefore requires exercise programs aimed at promoting a healthy lifestyle (Hara and Shimada, 2007). Available research is consensual and states that the process of aging is associated with loss of muscle strength (Candow, 2011; Clark et al., 2011; Hurley et al., 2011; Marzetti et al., 2009). Steib et al. (2010) used a meta-analysis to determine the dose-response relationship of resistance training to improve strength and function in older adults. The results showed that tests where different training intensities were compared showed strong effects of progressive resistance training on maximal strength in a dose-dependent manner, with high-intensity being more effective compared with moderate and low-intensity. The authors emphasized the need for more research to identify the effect of different training volumes and frequencies and the dose - response relationship for very old and frail populations (Steib et al., 2010).

From the available research, it seems also clear that the loss of maximum strength does not occur uniformly in all muscle groups (Clark et al., 2010). Two studies identified declines in strength of the elbow extensors and flexors lower than in knee extensors or flexors (Hughes et al., 2001; Lynch et al., 1999). Several researchers have associated this decline with the level of movement disorders (Sclicht et al., 2001; Sousa and Sampaio, 2005), which represents a major task for everyday living in the elderly. Thus, the strength of the lower limbs is probably the most dynamic muscle function and a valid indicator of functional capacity, being associated with independent living. In contrast, Rantanen et al. (1997) found a decline in isometric elbow flexion strength over 5 years but no changes in knee extension strength. These results may suggest that elderly subjects with high levels of mobility are more likely to have less pronounced loss of muscle strength when compared to elderly with poor functional status (Reid et al., 2008). Available research indicates that trainability is higher in less functional muscle groups (Clark et al., 2011; Fiatarone et al., 1990; 1994; Henwwod and Taaffe, 2005; Taaffe et al., 1999). Despite this, dose response research is needed to better understand the effects of strength training in elderly subjects and to better prescribe training programs. Therefore, the purpose of this study was to identify differences in maximum strength after an intense 12-week strength training program, contrasting muscle groups from upper limbs versus lower limbs.

## Material & Methods

### Sample

Ten healthy men (age 73±6 years old, body mass index (BMI) 23.4±1.2) were randomly selected from a group of sixteen responders to a local newspaper advertise. All participants were non-smokers, had no history of falls, no orthopaedic, neurologic, cardiac or pulmonary disorders that could restrict the activity and were moderately active. Moderate activity was defined as not participating in a planned fitness program more than three times/week, but more active than sedentary individuals. Additionally, none of the subjects had ever participated in a strength training program. All were from the rural north of Portugal, lived at home and could perform daily tasks without help. This program was approved by the Ethics Committee from the University of Trás-os-Montes e Alto Douro and all principles outlined in the Helsinki Declaration regarding research involving human subjects were followed.

### Strength training protocol

The subjects participated in a strength-training program consisting of 3 sessions each week for a period of 14 consecutive weeks. The average adherence rate was 95%, and none of the subjects participated in less than 90% of the sessions. The first 2 weeks prior to the training program were used as an acclimation period. The training sessions consisted of a circuit of 7 exercises: leg press, leg extension and leg curl for the lower limbs; bench press, lat pull down, shoulder press and arm curl for the upper limbs. All exercises were performed using variable resistance equipment (Image Sport Machines, Barcelona, Spain). All subjects completed the entire program with no dropouts. The intensity varied progressively between 50 and 80% of one-repetition maximum (1RM), 2–3 sets of 6–12 repetitions. Rest periods between sets were 30 seconds and 1 minute between exercises.

### Procedures

The program was initiated after the subjects completed an informed consent form and were told that they could withdraw, for whatever reason, at any time. The 1RM testing was carried by two experienced strength and conditioning trainers, following the standard guidelines (Kraemer and Fry, 1995; Niewiadomski et al., 2008). The 1RM testing occurred on a regular scheduled training day and constituted the workout for that day. In order to measure accurately the 1RM, the values were always obtained with 3 to 5 trials with an interval of 3 minutes between each trial. A total of four 1RM measures were taken on the first workout of week 1, 5 and 9 and the last workout of week 12. Training intensity was recalibrated to each new 1RM value. After this program and according to previously used procedures by Treuth et al. (1994), the analysis was carried out in 2 subgroups: LOWER (sum of average values of the three exercises for the lower limbs) and UPPER (sum of average values of the four exercises for the upper limbs). Percent variation was used for intra-group comparisons.

### Statistical Analyses

A two-factor repeated measures ANOVA was carried out for the statistical analysis with a muscle group and time as factors. All assumptions undergoing this statistical technique were tested and confirmed and Tukey HSD was used for post-hoc comparisons. Effect sizes (ES) were calculated to determine the magnitude of the effects and their interpretation was based on the following criteria: <0.20 = trivial, 0.20–0.59 = small, 0.60–1.19 = moderate, 1.20–2.0 = large, and >2.0 = very large (Hopkins, 2002). The level of significance was p≤0.05.

## Results

The results allowed to identify statistically significant differences in muscle group (F=8.1, p=0.006) and time effects (F=730.0 p=0.000), as well as a statistically significant interaction (F=4.4, p=0.014). In both groups (LOWER and UPPER), repeated measures ANOVA revealed statistically significant differences between baseline and all follow up 1RM values (Table 1).

There were small effects sizes in both LOWER and UPPER final values when contrasted with baseline (0.31 and 0.38, respectively). The most significant increase occurred in UPPER (81% vs. 66%). The percentage of accumulated gains in LOWER over the measurements taken was more tenuous compared with UPPER (Table 1). Additionally, the differences between LOWER and UPPER increased significantly during the training program (5, 9 and 15%, respectively).

## Discussion

The results from the current study showed higher relative gains in muscle groups of the upper limbs. The available literature is mainly focused on different changes in a muscle function with age (Gauchard et al., 2003; Henwwod and Taaffe, 2005), where it seems clear that there is a greater decline in the values of strength of these muscles which are used less frequently. Moreover, the loss of strength can reach values of around 10 to 15% per decade, mainly due to reduced muscle mass, specifically by reducing the number and size of muscle fibers type II (Gauchard et al., 2003). Thus, it seems clear that these losses of strength are mainly due to lack of stimulation.

In a longitudinal analysis, Rantanen et al. (1997) demonstrated that maintaining or increasing activity levels prevented or attenuated age-related strength losses. Impaired strength may result from a variety of factors, such as neuromuscular activation (Klass et al., 2007). Neuromuscular activation is the process by which excitation of motor neurons leads to force production in a population of muscle fibers. Each motor neuron and its associated muscle fibers constitute a motor unit, and the number and firing rate of recruited motor units are the major intrinsic determinant of muscular force (extrinsic factors such as muscle length and contraction velocity also influence force output). Weakness may directly result from impaired capacity of the nervous system to maximize motor unit recruitment and/or rate coding in agonist muscles (prime movers) or may be indirectly caused by poor intermuscular coordination or by excessive activation of antagonist muscles (which oppose the agonist). A number of age-related changes in the nervous system may contribute to impaired neuromuscular activation in older mobility– limited adults. For instance, loss of cortical projections to spinal motoneurons (Eisen et al., 1996), decreased inhibition between cerebral hemispheres (Peinemann et al., 2001), and reduced excitability of the corticospinal pathway (Oliviero et al., 2006) may lead to compromised ability to fully drive the motor pool.

Independent mobility is probably one of the last daily tasks to be lost with aging, especially in elderly subjects with independent living. Thereby, muscles of the lower limbs may be more elicited over the lifetime, and therefore have less strength losses. Therefore, it is perceived that their trainability is lower, in accordance with the results obtained in this study. In this context, the percentage gains were higher in muscle groups of the upper limbs, which probably would have a greater decline of strength, lower functionality and greater trainability. Although considering the strength of lower limbs, a valid indicator of functional capacity associated with mobility, it is also worth noticing that increased strength in upper limbs leads to improvements in balance and postural control, especially when performing motor tasks in motion or unsteadily, as the postural muscles tend to be the most affected with the losses (Gauchard et al., 2003; Sclicht et al., 2001; Sousa and Sampaio, 2005). Recently, Adamo et al. (2009) suggested that performing upper limb specific tasks might reduce age-related declines in proprioception.

Finally, from the results of this study, it was possible to verify that the differences between LOWER and UPPER significantly increased during the training program. This fact may be related to the intensity of the load gradually implemented (from 50 to 80% RM). Thus, it appears that for more intensity, the subjects responded with greater muscular adaptation (Sousa and Sampaio, 2005). Overall, studies that contrast the effectiveness of training programs for high versus low intensity confirm this fact (Sclicht et al., 2001).

## Conclusions

The studied group of elderly with independent living has earned higher maximum strength in the muscles of upper limbs. Furthermore, these gains tended to increase over time and with increasing intensity. This leads us to a conclusion and practical application that when planning specific exercise programs for healthy and independent elders, particular attention should be given to muscle groups located in the trunk and upper limbs. This is not only to elicit these muscle groups, but rather because they tend to be more debilitated. Also, the programs should be carefully designed, implemented and controlled in order to quantify the ratios of upper and lower limbs workload. Further research on this particular topic can be done by expanding the dose-response characteristics of the implemented programs, such as examining chronic responses from these populations, when comparing combinations of different training volumes and frequencies.

## Figures and Tables

**Table 1:** *Variation in 1RM (MEAN±SD) in LOWER and UPPER, effect size (ES) and the percentage gains from baseline (Δ%) following 4, 8, and 12 weeks of the strength-training program*.

	Baseline	After 4 weeks	After 8 weeks	After 12 weeks
LOWER (kG)	179 ± 27.9	201 ± 24.8*	222 ± 26.1*	282 ± 35.1*
ES (with baseline)	-	0.08	0.16	0.31
(Δ%)	-	14%*	25%*	66%*
UPPER (kG)	159 ± 26.4	187 ± 26.4*	214 ± 26.1*	270 ± 32.2*
ES (with baseline)	-	0.10	0.21	0.38
(Δ%)	-	19%*	34%*	81%*
LOWER-UPPER (Δ%)		5%	9%	15%

* *Denotes significant differences from previous 1RM test*.
